# Supplementary material for: Mitoregulin Promotes Cell Cycle Progression in Non-Small Cell Lung Cancer Cells
Source: Int J Mol Sci. 2025 Feb 24;26(5):1939. doi: 10.3390/ijms26051939 (PMC11899852; doi:10.3390/ijms26051939)
Supplement: Supplementary file 1 [file ijms-26-01939-s001.zip › Supplement Figures S1 to S4.pdf]

International Journal of
Molecular Sciences

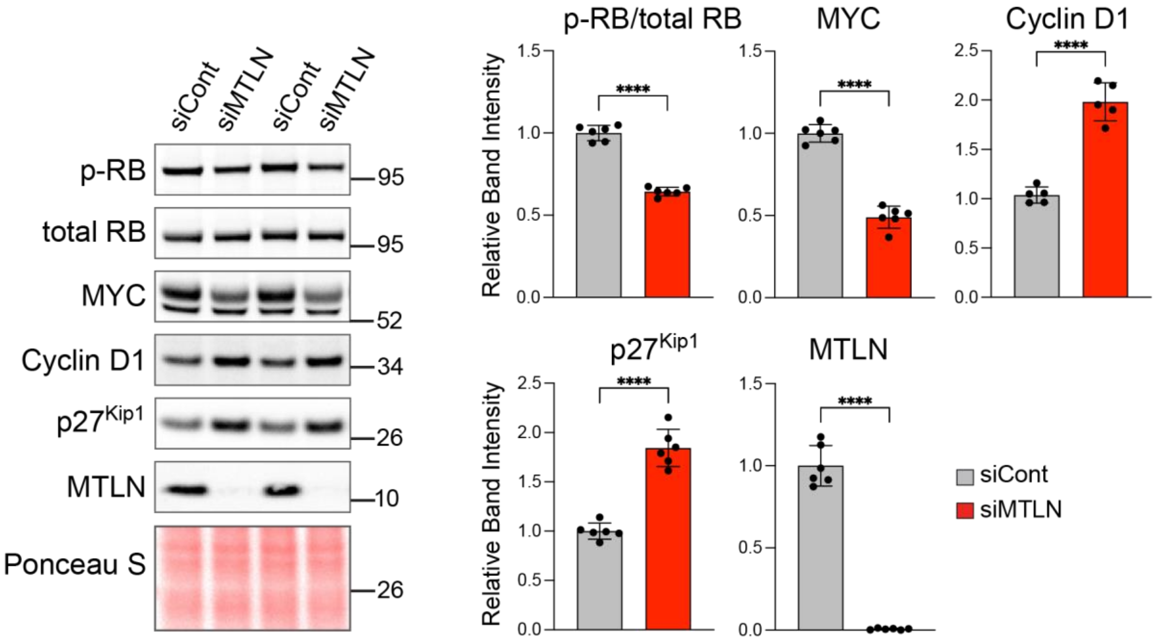

Figure S1. MTLN silencing in H1299 lung adenocarcinoma cells imparts a G1 cell cycle arrest profile. Cell lysates harvested 48 h after siCont or siMTLN treatment were assayed for proteins by Western blot. Bars show mean ± SD of relative band intensities. Phospho-RB band intensities were determined relative to total RB band intensity in the same lane; other protein band intensities were normalized to Ponceau S in the same lane ($n = 5$ or 6 per group) ****$p < 0.0001$ by unpaired T-test.

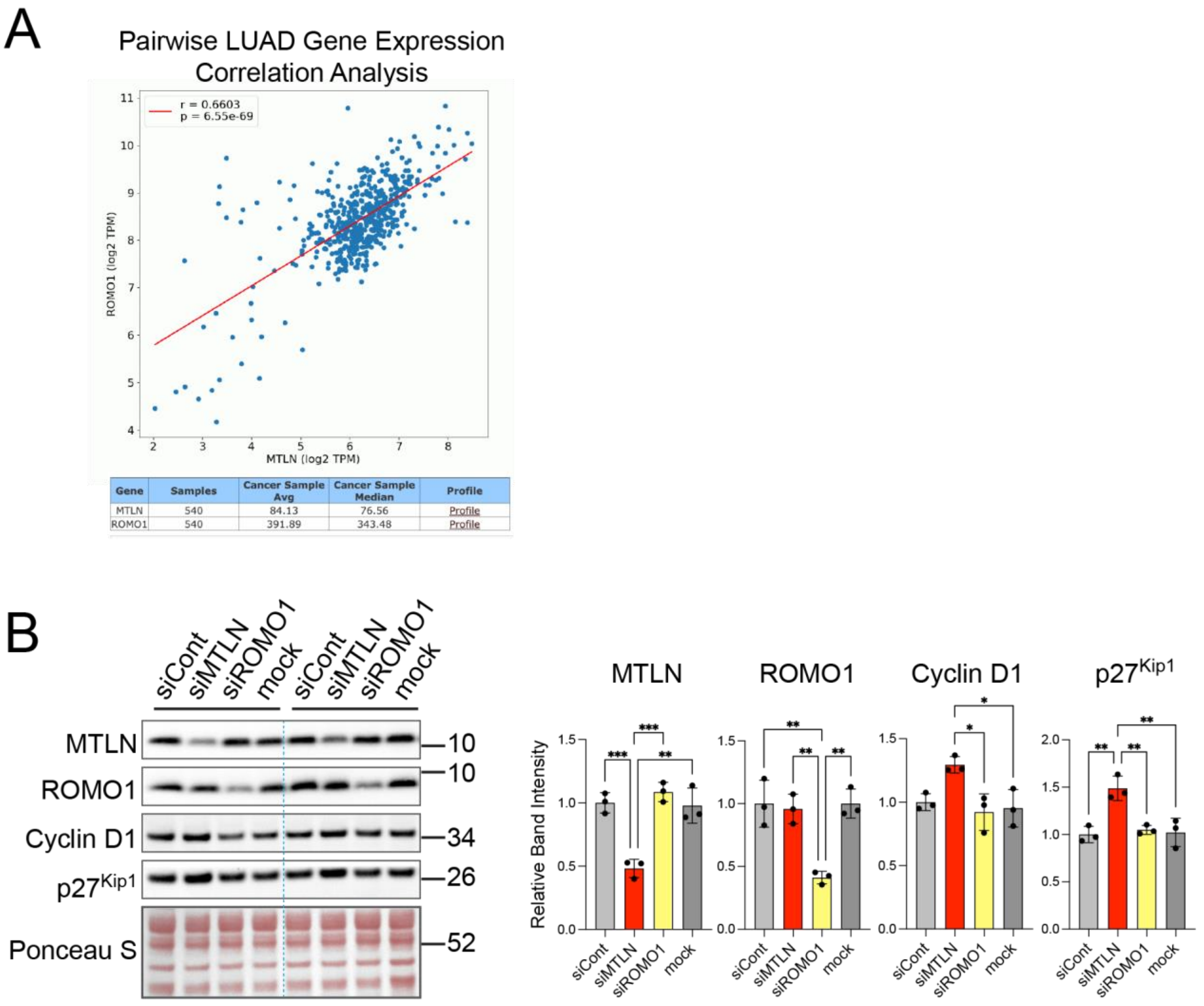

Figure S2. Similarities and difference between MTLN and ROMO1 in relation to LUAD. (**A**) High gene expression correlation of MTLN and ROMO1 in lung adenocarcinoma (LUAD). Pair-wise Gene Expression Correlation was performed using the online tool on the OncoDB website. Scatter plot shows log2 TPMs for MTLN and ROMO1 gene expression ($n = 540$) (**B**) Altered $p27^{Kip1}$ and cyclin D1 protein abundance after MTLN- but not ROMO1- KD in A549 cells. Cell lysates harvested 48 h after siCont, siMTLN, siROMO1, or mock treatment were assayed for protein levels by Western blot. Histograms show mean ± SD of relative band intensities, normalized to Ponceau S within the same lane. (n = 3 per group) *$p < 0.05$, **$p < 0.01$, ***$p < 0.001$ by 1-way ANOVA with multiple comparisons.

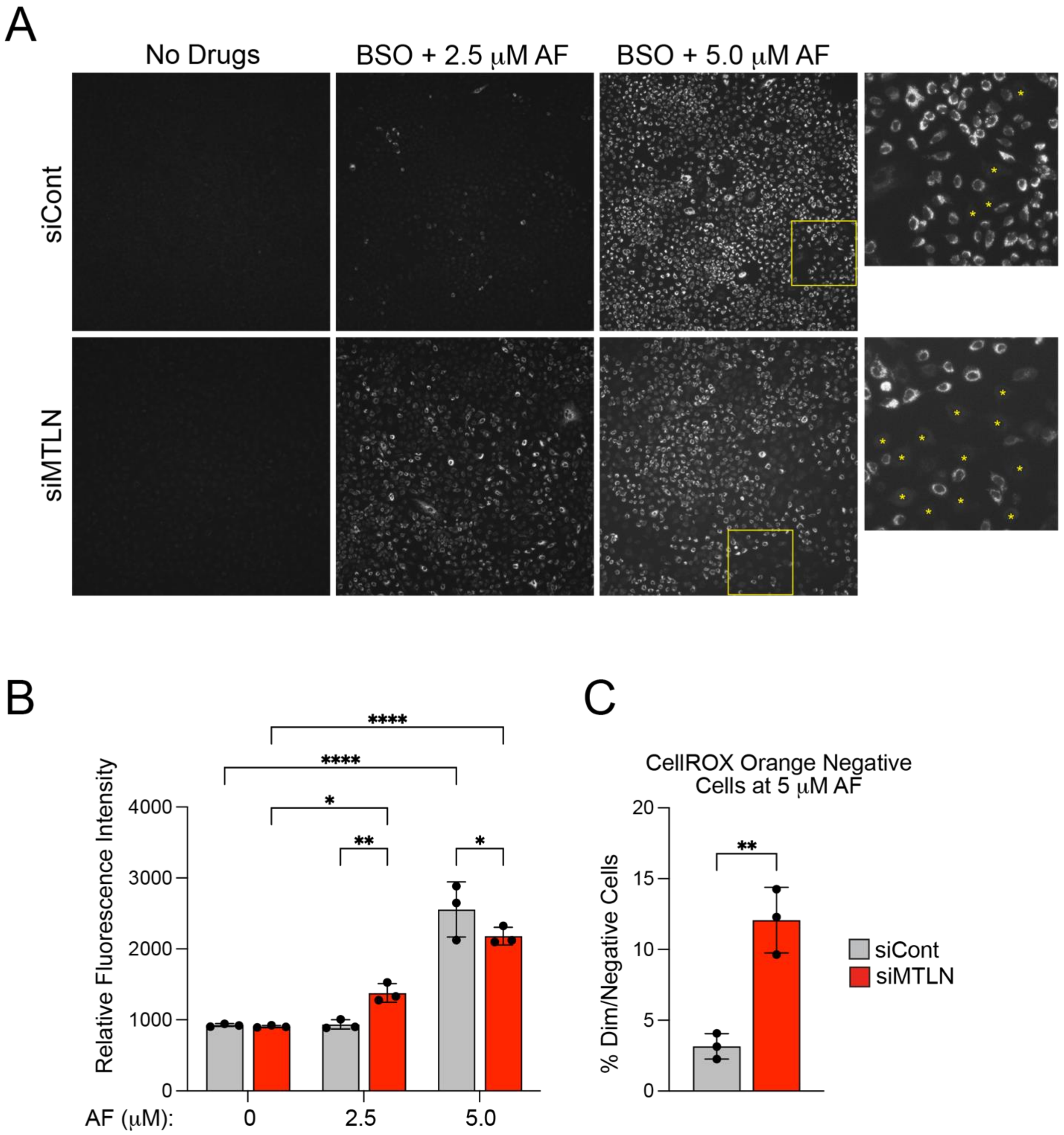

Figure S3. BSO plus auranofin treatment increases ROS as determined by CellROX Orange fluorescence. At 48 h post siMTLN or siCont treatment, BSO (100 μM) was added to A549 cell cultures. Twenty-four hours later, BSO-treated cultures were exposed to 2.5 μM or 5.0 μM AF for 3 h, loaded with CellROX Orange ROS sensor and live fluorescent images captured. (**A**) Representative images from each group are shown. Zoomed areas from the 5 μM AF images displayed on the right illustrate increased proportion of non-fluorescent/dim cells (marked with yellow asterisks) in siMTLN compared to siCont groups. (**B**) Histogram shows mean ± SD CellROX Orange fluorescence intensities of images collected from each group, treated as described in (A). ($n = 3$) *$p < 0.05$, **$p < 0.01$, ****$p < 0.0001$ by 2-way ANOVA with multiple comparisons. (**C**) Histogram shows the mean ± SD proportion of non-fluorescent/dim cells after 5 μM AF ($n = 3$) **$p < 0.01$ by unpaired t-test.

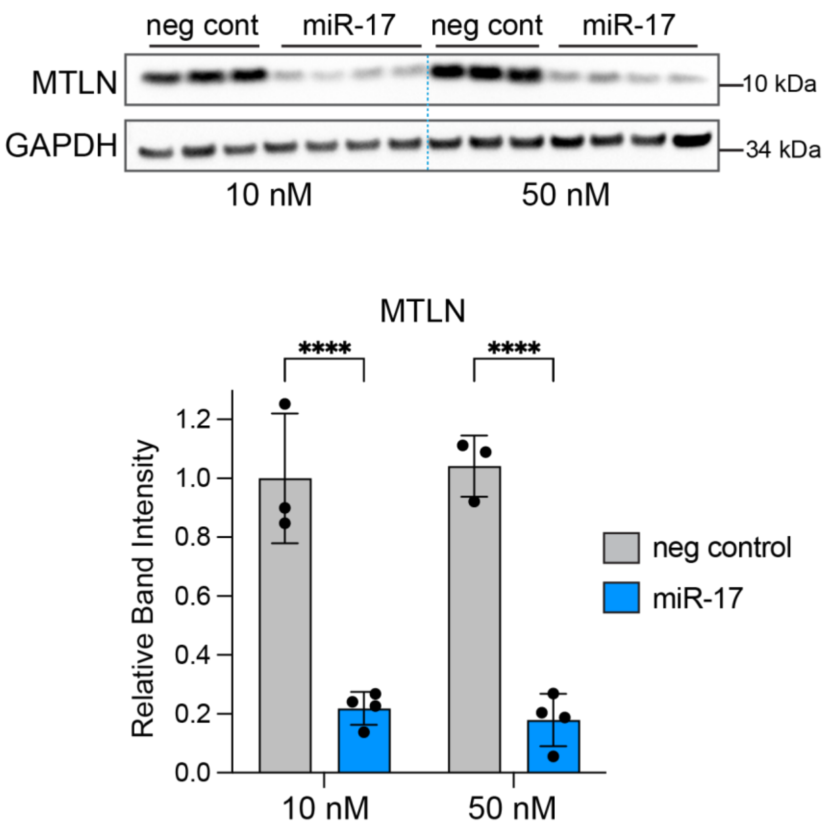

Figure S4. MTLN is strongly suppressed by miR-17 mimic. A549 cells were transfected with negative control or miR-17 (Hsa-miR-17-5p) miRNA mimic at 10 nM and 50 nM and 48 h later cell lysates were examined by western blot for MTLN protein levels. MTLN band intensities were normalized to GAPDH band intensities in the same lane. Bars show the mean ± SD, relative to low-dose negative control (n = 3 or 4 per group) ****$p < 0.0001$ by 2-way ANOVA with multiple comparisons.
